# Clinical Course and Perinatal Outcomes of Pregnant Women with COVID-19 in Central Greece: A Prospective Cohort Study

**DOI:** 10.3390/diseases14050178

**Published:** 2026-05-19

**Authors:** Christos Donoudis, Antonios Garas, Sotirios Sotiriou, Ioannis Pantazopoulos, Athanasios Pagonis, Eleni Zachari, Nikoletta Daponte, George Syrogiannopoulos, Ioanna Grivea, Alexandros Daponte

**Affiliations:** 1 Department of Obstetrics & Gynaecology, University of Thessaly, 41110 Larissa, Greece; 2 Department of Embryology, University of Thessaly, 41110 Larissa, Greece; 3 Department of Respiratory Medicine, University of Thessaly, 41100 Larissa, Greece; ipantazop@uth.gr (I.P.);; 4 Intensive Care Unit, University Hospital of Larissa, 41110 Larissa, Greece; 5 Department of Pediatrics, University of Thessaly, 41110 Larissa, Greece; syrogian@otenet.gr (G.S.);

**Keywords:** COVID-19, pregnancy, clinical outcomes, perinatal outcomes, vaccination, ischemia-modified albumin

## Abstract

Background: During the COVID pandemic increased rates of intensive care unit (ICU) admission, mechanical ventilation, caesarean delivery, and preterm birth among women with SARS-CoV-2 infection in pregnancy were recorded. Purpose: This study describes the clinical course and perinatal outcomes of pregnant women with COVID-19 across pre- and post-vaccination periods. Methods: This study included all pregnant women with confirmed SARS-CoV-2 infection who subsequently delivered at the University General Hospital of Larissa between March 2020 and May 2023. Demographics, comorbidities, gestational age at infection and at delivery, COVID-19 symptoms, need for hospitalization, obstetric complications, mode of delivery, and neonatal outcomes were documented. An assessment of ischemia-modified albumin (IMA) was performed in a subset of women. Results: A total of 327 women (including 14 twin gestations) were recorded. Most women experienced mild disease while a minority required hospital admission, or intensive care (1.8 and 0.3% for the studied population, respectively). Fever and upper respiratory symptoms predominated, while radiologic evidence of pneumonia was rare. Overall preterm birth (<37 weeks) occurred in 13% of pregnancies and caesarean section in about two thirds of deliveries. Neonatal outcomes were favorable, with low rates of neonatal intensive care unit (NICU) admission and no early neonatal deaths. IMA values were higher during acute infection and declined towards recovery. Conclusion: Pregnant women with COVID-19 in Central Greece had predominantly mild clinical courses and excellent perinatal outcomes. IMA may represent a biologically plausible marker of disease activity, but further studies are needed.

## 1. Introduction

The first months of the COVID-19 pandemic were marked by profound clinical uncertainty and a sense of health-system panic. Early reports from China and later from Europe and the United States suggested that pregnant women infected with SARS-CoV-2 might be at increased risk for severe respiratory disease, admission to the ICU and invasive ventilation compared with non-pregnant women of reproductive age [1,2]. At the same time, clinicians worried about the risk of miscarriage, congenital anomalies, preterm birth, fetal growth restriction and vertical transmission [3,4]. Hospitals rapidly re-organized obstetric services, implementing triage systems, separate COVID-19 delivery units and restrictions on birth companions and postnatal visits.

Moreover, the emergence and global spread of new and renewed infectious diseases are increasingly understood as being entwined with social conditions such as disparity, discrimination, and structural violence. This may necessitate a “syndemic” approach, which conceptualizes COVID-19 not as an isolated biological event but as a synergistic interaction among coexisting health conditions and social environments that produce an excess burden of disease [5].

Large observational cohorts and systematic reviews gradually provided a more detailed picture. Meta-analyses have confirmed an increased risk of ICU admission, mechanical ventilation, and iatrogenic preterm birth among pregnant women with COVID-19, but also reported low rates of stillbirth and neonatal death in contemporary practice [1,3,6,7]. The introduction of effective vaccines and evidence supporting vaccination during pregnancy markedly improved maternal outcomes, reducing the incidence of severe disease and critical illness [8,9,10].

In Greece, the Hellenic Society of Obstetrics and Gynecology (HSOG/ΕΜΓΕ) issued stepwise guidance on the management of pregnant women with COVID-19 and on SARS-CoV-2 vaccination during pregnancy and lactation [11,12]. These national recommendations, coupled with international guidelines from professional societies and the World Health Organization, informed local protocols at tertiary centers [13].

Despite the accumulating international data, there is limited published evidence describing real-world maternal and perinatal outcomes from Greek populations throughout the entire course of the pandemic, including the later period when vaccination was widely available. Furthermore, only a few studies have examined potential biomarkers that might help identify women at risk of deterioration [14,15,16,17]. Ischemia-modified albumin (IMA) is an oxidative stress marker that has been associated with disease severity in non-pregnant COVID-19 cohorts [18,19,20], but its behavior during pregnancy-associated infection is largely unexplored.

The University Hospital of Larissa serves as a tertiary referral center for the 5th Health Region (Thessaly and Central Greece), providing clinical coverage for a catchment population of approximately 1.2 million inhabitants. Approximately, 1000 to 1200 births are performed in the Obstetrics and Gynecology Department. The primary objective of this study was to describe the clinical characteristics and perinatal outcomes of pregnant women with COVID-19 in Central Greece across all pandemic phases and possible associations of measured biomarkers. Secondary objectives were to explore the association between gestational age at infection and pregnancy outcome and to provide a preliminary descriptive analysis of IMA levels in a subset of women.

## 2. Materials and Methods

### 2.1. Study Design and Setting

This was a prospective observational cohort study conducted at the University General Hospital of Larissa, a tertiary referral center for Central Greece with a high-volume obstetric unit and neonatal intensive care unit. The study period extended from 1 March 2020, shortly after the first national lockdown, until 31 May 2023, when the pandemic state of emergency was officially lifted.

### 2.2. Study Population and Inclusion Criteria

All pregnant women with confirmed SARS-CoV-2 infection (positive RT-PCR or rapid antigen test) at any time during pregnancy who subsequently delivered at our institution were eligible. Women with suspected but unconfirmed infection were not included. Re-infections were recorded, but each pregnancy contributed only one index episode. Multiple gestations were included.

### 2.3. Data Collection and Registry

At the onset of the pandemic, a structured registry was designed to ensure systematic and prospective data collection. Data were extracted from antenatal clinic records, hospital admissions, delivery notes and neonatal charts, and directly from the patients with the use of a questionnaire and were entered into anonymized Excel worksheets. The registry captured:

Maternal baseline characteristics: age, body mass index, parity, multiple pregnancy, pre-existing medical conditions (including thyroid disease, diabetes, autoimmune disorders and thrombophilias).

Obstetric history and complications: prior preterm birth, miscarriage, cervical cerclage, placenta praevia, threatened miscarriage, vaginal bleeding in the current pregnancy, hypertensive disorders, gestational diabetes and other pregnancy complications.

Gestational age at infection and at delivery: gestational week at first positive test, trimester of infection, interval between infection and delivery. Special attention was given to infections before 20 weeks’ gestation, in line with the hypothesis that early infection might influence the risk of preterm birth, pre-eclampsia, fetal growth restriction or low birth weight.

Clinical presentation of COVID-19: presence and type of symptoms (fever, cough, dyspnea, anosmia, gastrointestinal symptoms), asymptomatic cases, need for chest imaging and radiologic findings.

Severity and treatment: outpatient management versus hospitalization; need for oxygen therapy, high-flow support or mechanical ventilation; admission to high-dependency or intensive care units; use of anticoagulation, corticosteroids, antiviral therapy or tocilizumab according to evolving local and national protocols [11,12,21].

Delivery data: gestational age at delivery, onset of labour (spontaneous, induced, elective caesarean), indication for caesarean section, complications during labour, postpartum hemorrhage.

Neonatal outcomes: birth weight, sex, Apgar scores, need for resuscitation, NICU admission, early neonatal complications, and SARS-CoV-2 testing when performed.

Biomarkers (exploratory): serological measurements, including IMA values at delivery and, for a small subset of women, IMA values during hospitalization, as well as C-reactive protein, liver enzymes and hematologic indices.

### 2.4. Management Protocol

Clinical care was provided according to hospital algorithms that were regularly updated to align with evolving evidence and HSOG guidelines [11,12]. Key elements included:

Initial triage and diagnosis of symptomatic pregnant women in dedicated COVID-19 areas.

Risk stratification based on symptoms, comorbidities, gestational age and oxygen saturation.

Outpatient surveillance for mild disease through remote or in-person follow-up, supplemented by standardized education on red-flag indicators (worsening dyspnea, persistent fever, reduced fetal movements).

Inpatient care for women with moderate or severe disease, hemodynamic instability or obstetric complications, with multidisciplinary input from obstetricians, respiratory physicians and intensivists.

Delivery planning: timing and mode of delivery were based on obstetric indications and maternal/fetal status rather than SARS-CoV-2 positivity alone, in accordance with international recommendations [6,8,22].

Neonatal management: delayed cord clamping and rooming-in were encouraged when clinically appropriate; breastfeeding was supported with infection-prevention measures. Neonates were tested for SARS-CoV-2 according to national policies and clinical indications.

### 2.5. Ethical Approval

The study protocol was approved by the Scientific Council of the University General Hospital of Larissa (Protocol No. 12263/13 April 2021). All data were anonymized prior to analysis. The study was conducted in accordance with the Declaration of Helsinki.

### 2.6. Statistical Analysis

Given the exploratory nature of this interim report, the present analysis is primarily descriptive. Statistical analyses were conducted using R software (Version 4.5) (R Core Team; R: A Language and Environment for Statistical Computing; R Foundation for Statistical Computing, Vienna, Austria). Continuous variables are reported as mean ± standard deviation when normally distributed and as median with interquartile range when normality assumptions were not met. Normality was evaluated using the Shapiro–Wilk test. Categorical variables are summarized using counts and percentages. Differences in mean values between groups were analyzed using Student’s *t*-test. Levene’s test was used to assess the homogeneity of variance.

## 3. Results

### 3.1. Baseline Characteristics

A total of 327 women with confirmed SARS-CoV-2 infection during pregnancy delivered at our institution during the study period. Fourteen pregnancies were twin gestations. Mean maternal age was approximately 32.2 years. A considerable proportion (*n* = 66; 20.2%) of women had at least one pre-existing medical condition, most commonly thyroid disease (*n* = 30; 9.1%), autoimmune disorders (*n* = 17; 5.1%) or heterozygosity for thrombophilia markers (*n* = 2; 0.6%) (Table 1).

### 3.2. Gestational Age at Infection

**Figure 1:**
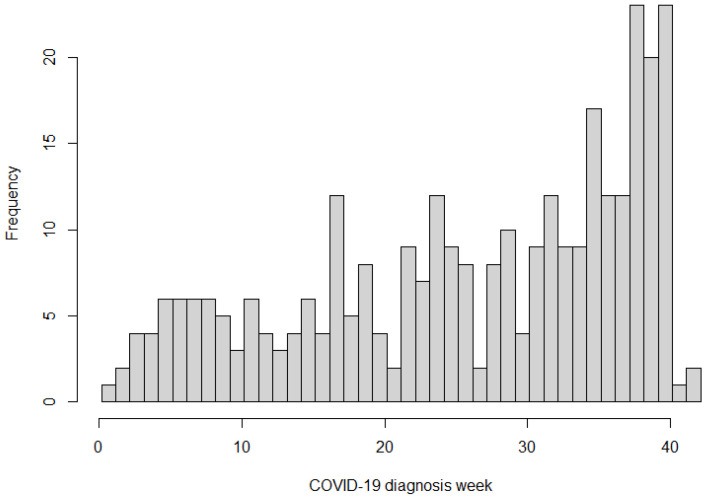
Weekly Distribution of COVID-19 Diagnoses.

According to Figure 1, infections occurred across all trimesters, with a clustering in the second and third trimesters, reflecting community transmission patterns. A smaller subgroup acquired infection before 20 weeks’ gestation. These early infections were followed closely because of theoretical concerns regarding miscarriage, placental dysfunction, pre-eclampsia, fetal growth restriction and preterm birth.

### 3.3. Clinical Presentation and Severity

The clinical course was predominantly mild. Fever was the most frequent symptom (*n* = 188; 57.5%), followed by nasal congestion (*n* = 136; 41.6%), cough (*n* = 123; 37.6%), taste disorder (*n* = 77; 23.5%), sore throat (*n* = 64; 19.6%), and upper respiratory complaints (*n* = 51.5%). Vaccinated patients remained without fever in a significantly higher level (*p* < 0.05) compared to unvaccinated ones (42.1 and 25.3% for vaccinated and unvaccinated women, respectively) (Table 2). A notable proportion of women remained asymptomatic (*n* = 49; 15%) and were diagnosed during routine screening before admission. Very few women developed radiologically confirmed pneumonia (*n* = 4; 1.2%); chest imaging remained normal in the vast majority of those who underwent radiography or computed tomography. Only a small subset required hospitalisation (*n* = 6; 1.8%), and only one needed intensive care and mechanical ventilation (*n* = 1; 0.6%).

### 3.4. Obstetric Complications

The registry included a detailed catalog of obstetric complications that occurred during pregnancy, such as threatened miscarriage (*n* = 7; 2.1%) and vaginal bleeding in the first trimester (*n* = 24; 7.3%), cervical insufficiency requiring cerclage (*n* = 5; 1.5%), placental abnormalities (*n* = 1; 0.3%), hypertensive disorders (*n* = 4; 1.2%), and gestational diabetes (*n* = 70; 21.4%). Each complication was carefully documented with corresponding frequencies. Overall, the rates of major obstetric complications—including pre-eclampsia (*n* = 1; 0.3%), significant placental pathologies (*n* = 4, 1.2%) and severe haemorrhage (*n* = 1, 0.3%) were low and within the range expected for a tertiary obstetric population.

### 3.5. Mode and Timing of Delivery

Most women delivered at term. Overall, preterm birth (<37 weeks) occurred in 50 (15.2%) pregnancies, with very preterm birth (<32 weeks) being rare (*n* = 7; 2.1%). A review by Saad et al. [23] shows similar results with 12.69% of the patients with COVID having preterm deliveries. The overall caesarean section rate was around two-thirds of deliveries (*n* = 214; 65.4%). Many caesarean sections reflected pre-existing high institutional rates and obstetric indications rather than acute deterioration from COVID-19. Among women who were still SARS-CoV-2 positive at the time of delivery, the caesarean rate remained high but was not associated with a parallel increase in neonatal morbidity.

### 3.6. Neonatal Outcomes

Median birth weight was (3.13 kg; IQR = 0.64) appropriate for gestational age in most cases, and the incidence of small-for-gestational-age infants was low (*n* = 8; 2.4%). Apgar scores at 1 and 5 min were reassuring in the vast majority of neonates (Median = 9; IQR = 0, min = 7, max = 10). NICU admission was necessary for a small proportion of infants (*n* = 10; 3.1%), mainly due to prematurity or non-COVID-related reasons. No early neonatal deaths were recorded. Four cases (1.2%) of confirmed vertical transmission with no clinical neonatal disease were observed. There were no significant correlations of birth weight and the occurrence of COVID infection in the studied population of women with more than one late term pregnancies.

### 3.7. Exploratory Biomarker Analysis: Ischemia-Modified Albumin

**Figure 2:**
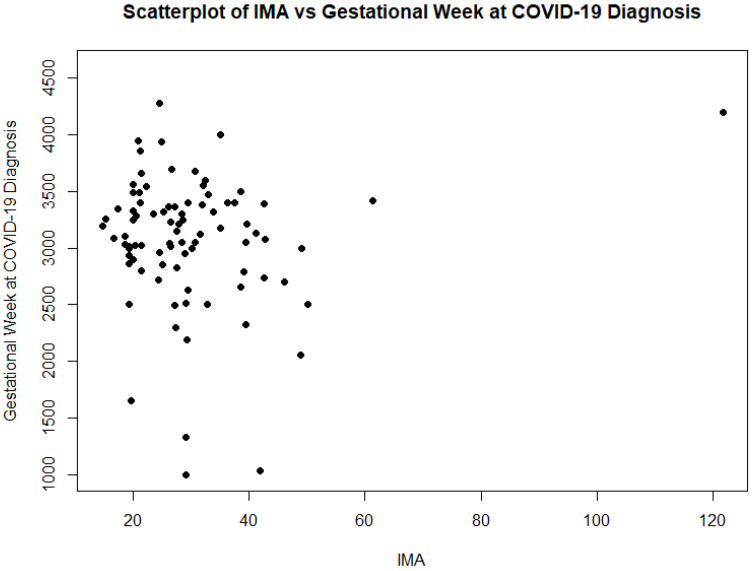
A Spearman’s rank correlation analysis showed a statistically significant positive association between IMA and the gestational week at COVID-19 diagnosis (rho = 0.25, *p* = 0.02).

IMA values at delivery were available for 86 (26.3%) women, while a smaller subgroup had measurements during hospitalisation (*n* = 5; 1.5%). These samples were distributed across the initial stages of the pandemic, specifically occurring during 2021 and 2022 (32 patients in 2021 and 54 in 2022, respectively). Values showed considerable inter-individual variability. Overall, IMA levels tended to be higher during the acute phase of moderate or severe illness and displayed a declining trend towards recovery and delivery. In one critically ill patient requiring mechanical ventilation, IMA remained substantially elevated during the period of respiratory failure and decreased in parallel with clinical improvement. However, due to the limited number of severe cases and the absence of formal statistical modelling, these observations should be considered hypothesis-generating. IMA elevation was significantly higher to unvaccinated women compared to vaccinated ones (*p* < 0.05) (32.21 and 25.19, respectively). A statistically significant correlation between IMA and the gestational week at COVID-19 diagnosis was documented (*p* < 0.05) (Figure 2).

A weak negative correlation was identified between IMA levels and the year of delivery (Pearson r = −0.22, *p* = 0.039), indicating a general downward trend in oxidative stress markers from 2021 to 2022. Despite this trend, the difference between the two years did not reach statistical significance in non-parametric analysis (Mann–Whitney *p* = 0.066), suggesting that while mean values appeared lower in the later phase of the pandemic, the variation is not definitively established within the current sample.

## 4. Discussion

This prospective cohort provides a comprehensive real-world overview of the clinical course and pregnancy outcomes of women with COVID-19 who delivered in a tertiary center in Central Greece from the start of the pandemic until May 2023. The principal finding is that, despite initial fears and the unprecedented health-system stress of the early waves, most pregnant women experienced mild disease and favorable obstetric and neonatal outcomes.

Our results align with large systematic reviews and meta-analyses that have reported increased relative risks of ICU admission and preterm birth among infected pregnant women, but low absolute rates of maternal mortality and perinatal death in modern practice but the results were not statistically corroborated [1,3,6,7]. Early in the pandemic, cohorts of hospitalized pregnant women from the United Kingdom, the United States and France described high burdens of severe respiratory disease and medically indicated preterm delivery in the context of maternal decompensation [2,24,25]. As experience increased, along with the introduction of vaccination and enhanced supportive care, multicenter studies later showed a trend toward milder disease and improved outcomes [4,10,26]. Our findings from Central Greece fit within this evolving global narrative.

The high caesarean section rate in our cohort is comparable to that reported in several early COVID-19 series and meta-analyses [1,6]. It likely reflects a combination of underlying institutional practice patterns, clinician preference during the period of greatest uncertainty, and obstetric indications. Importantly, the high caesarean rate was not associated with an excess of adverse neonatal outcomes. Most neonates had satisfactory Apgar scores, appropriate birth weights and short postnatal hospital stay, similar to findings from large registries such as COVIPREG and international multicenter collaborations [4,25].

Beyond the clinical data, this study highlights the emergence of a syndemic involving the physiological manifestations of SARS-CoV-2 and the socio-technical environment of the healthcare system. The “institutional obstetric panic” of the early pandemic characterized by profound clinical uncertainty and the rapid reorganization of services likely contributed to the high baseline of caesarean deliveries (65.4%) that persisted even as clinical outcomes remained favorable.

The seasonality and trimester distribution of infection in our population mirror community transmission patterns and vaccination rollout in Greece. Notably, infections occurring in early gestation did not translate into a clear increase in severe pregnancy complications such as pre-eclampsia, fetal growth restriction or major congenital anomalies, although numbers were limited and formal adjustment for confounders is pending. This is consistent with several recent studies that have not demonstrated a strong teratogenic effect of SARS-CoV-2 but have highlighted potential associations with placental lesions and subtle growth abnormalities in some settings [13,27,28].

Our exploratory analysis of IMA adds a novel, though preliminary, layer to the literature. IMA has been studied as a marker of oxidative stress and ischemia in cardiovascular disease and, more recently, in COVID-19, where elevated levels have been associated with severe pneumonia, coagulopathy and poorer prognosis [18,19,20]. In the present cohort, IMA trajectories appeared biologically plausible—higher during severe or critical illness and decreasing as the clinical course improved. Moreover, considering IMA as a prognostic biomarker for the clinical outcome of COVID-19, the reduced levels in vaccinated women show the beneficial effects of vaccination. Future work with larger samples, control groups and multivariable analysis is required to determine whether IMA offers independent prognostic information during pregnancy.

Several factors likely contributed to the favorable outcomes observed. First, structured clinical pathways for triage, monitoring and delivery planning were implemented early and updated in line with HSOG and international recommendations [8,9,11,12,22]. Second, access to vaccination and increasing uptake among pregnant women and those planning pregnancy would be expected to reduce the incidence of severe disease, as consistently shown in multiple studies and meta-analyses [8,9,10]. Third, interdisciplinary collaboration between obstetricians, respiratory physicians, intensivists and neonatologists facilitated timely escalation of care when necessary.

The main strengths of this study are its prospective design, inclusion of all infected women delivering at a large referral center over an extended period covering multiple pandemic waves, and the rich clinical detail captured in the registry. The study also has limitations. It represents the experience of a single tertiary hospital, which might limit its general applicability. Some women with COVID-19 may have delivered in other facilities and are therefore not captured. The present analysis is largely descriptive and interim; formal multivariable modelling by a biostatistician is still pending. Finally, the exploratory IMA component involves a limited number of severe cases.

Despite these limitations, our findings provide reassuring evidence regarding maternal and neonatal outcomes of COVID-19 in pregnancy in Central Greece and underscore the value of structured protocols and vaccination. They also highlight avenues for future research, including more detailed evaluation of biomarkers such as IMA, long-term neurodevelopmental follow-up of children exposed in utero, and robust comparisons between different pandemic waves and viral variants.

## 5. Conclusions

In this prospective cohort of 327 pregnant women with COVID-19 who delivered at a tertiary center in Central Greece from March 2020 to May 2023, the clinical course was predominantly mild and perinatal outcomes were excellent. Preterm birth and NICU admission rates were low, four confirmed cases of no clinically significant vertical transmission occurred and no maternal deaths were reported. These reassuring results likely reflect the combined effects of structured, guideline-based care and widespread SARS-CoV-2 vaccination. Exploratory observations suggest that IMA may follow disease activity, but its prognostic utility in pregnancy remains to be defined. Continued surveillance and more detailed analyses are required to fully understand the long-term implications of COVID-19 in pregnancy.

## Figures and Tables

**Table 1:** Socio-demographic Data.

Age	
mean (SD)	32.2 (6.50)
Smoking	
No	248 (75.8%)
Yes	31 (9.5%)
Missing	2 (0.6%)
Height	
mean (SD)	166 (5.84)
BMI_before_PG	
mean (SD)	24.8 (6.05)
BMI_at_delivery	
mean (SD)	28.9 (5.12)
Health Issues Prior to Pregnancy	
No	261 (79.8%)
Yes	66 (20.2%)

**Table 2:** Main Clinical Symptoms during COVID-19.

Initial symptoms fever	
No	139 (42.5%)
Yes	188 (57.5%)
Initial symptoms cough	
No	204 (62.4%)
Yes	123 (37.6%)
Initial symptoms taste disorders	
No	250 (76.5%)
Yes	77 (23.5%)
Initial symptoms Sore throat	
No	263 (80.4%)
Yes	64 (19.6%)
Initial symptoms nasal congestion	
No	191 (58.4%)
Yes	136 (41.6%)
Initial symptoms other	
No	278 (85.0%)
Yes	49 (15.0%)

## Data Availability

The original contributions presented in this study are included in the article. Further inquiries can be directed to the corresponding authors.
